# Incidental Otolaryngologic Pathology Noted Upon Esophagogastroduodenoscopy: Case Series and Review of Literature

**DOI:** 10.7759/cureus.11866

**Published:** 2020-12-03

**Authors:** Eric J Macdonald, Benjamin A Lerner, Michael Z Lerner

**Affiliations:** 1 Otolaryngology, Albert Einstein College of Medicine, Bronx, USA; 2 Medicine, Section of Digestive Disease, Yale School of Medicine, New Haven, USA; 3 Otolaryngology, Yale School of Medicine, Greenwich, USA

**Keywords:** esophagogastroduodenoscopy (egd), endoscopy, gastroenterology, otolaryngology, referral and consultation, incidental findings

## Abstract

Objectives: We report and analyze eight cases in which patients were referred from gastroenterology (GI) to otolaryngology following esophagogastroduodenoscopy (EGD). We aim to provide specific examples of head and neck pathology encountered by gastroenterologists during upper endoscopy.

Methods: A series of eight cases between 2016 and 2019 were analyzed by chart review. In each case, otolaryngology consultation was requested after an abnormality was noticed by a gastroenterologist during EGD. Subsequent laryngoscopy or bronchoscopy was performed in all cases allowing for image comparison. Select images comparing EGD to laryngoscopy findings are included as well as a literature review concerning the nature of communication between the two specialties.

Results: Eight adult patients were referred to otolaryngology for abnormalities noted by a gastroenterologist during EGD at the following anatomic sites: soft palate (n=1), base of tongue (n=2), glottis (n=3), and interarytenoid mucosa (n=1). Additionally, a potential airway foreign body was noted on EGD which was ultimately determined to represent normal subglottic anatomy by bronchoscopy. Some 5/8 (63%) cases were considered true pathology while 3/8 (37%) represented normal anatomy or anatomic variants upon subsequent otolaryngologic evaluation.

Conclusions: There is minimal literature regarding the nature of referrals from GI to otolaryngology following EGD. Our findings suggest that EGD offers a unique opportunity for early detection of otolaryngologic pathology. However, certain inter-specialty anatomic knowledge gaps were noted which contributed to occasional unnecessary referrals, procedures, and associated patient anxiety. We hope that the results of this study can inform future research aimed at improving communication and collaboration between the two specialties.

## Introduction

This research was previously presented at the Fall Voice Conference in Dallas, Texas on October 17th, 2019. 

The larynx and hypopharynx are both common sites for head and neck cancers in which patient survival depends greatly on early diagnosis [1]. The often vague and benign initial presenting symptoms of these cancers, such as cough and sore throat, make early detection particularly challenging [2]. Furthermore, laryngopharyngeal cancer morbidity remains high due to compromised function of essential aerodigestive and phonatory structures [3]. Jacobson et al. suggest that the cost of head and neck cancers disproportionately burdens the US healthcare system when compared to other malignancies [4], yet despite this, there are no standardized screening tests for laryngopharyngeal cancers [1].

In many patients the first visualization of the laryngopharynx occurs incidentally during the millions of esophagoduodenoscopies (EGDs) performed annually [1, 5]. Currently, upper endoscopy guidelines do not recommend examination or photo-documentation of the larynx, pharynx, or trachea during EGD [6]. However, recent findings suggest that evaluation of these regions during EGD can be accomplished both safely and quickly [1].

The objective of this case series and review of the literature is to further investigate the nature of referrals from gastroenterology (GI) to otolaryngology following EGD. We aim to provide specific examples of head and neck pathology encountered by gastroenterologists during upper endoscopy.

## Materials and methods

This study was reviewed and approved by the Albert Einstein College of Medicine’s Institutional Review Board. All subjects included in this study were referred to the otolaryngology department after abnormal findings during EGD. Due to the retrospective nature of this study, the gastroenterologists received no special instructions during these EGDs. Images were selected for inclusion in this paper based on educational value, image quality, and ability to reasonably compare EGD and laryngoscopy images.

## Results

A series of eight cases between 2016 and 2019 referred to an academic otolaryngology department were reviewed. A summary of the data is provided in Table 1. Our subjects’ ages ranged from 38 to 70 years with a mean age of 57.5 years at the time of EGD. Some 5/8 patients (63%) were female. EGD revealed abnormalities at the following anatomic sites: soft palate (n=1), base of tongue (n=2), glottis (n=3), interarytenoid mucosa (n=1), and lastly a possible airway foreign body. Some 5/8 cases (63%) were diagnosed as true pathology upon subsequent otolaryngologic evaluation, while 3/8 (38%) represented normal anatomy or anatomic variants. 

**Table 1:** Summary of eight cases referred from GI to ENT after abnormal findings during EGD. ^a^Patient did not receive any imaging during otolaryngology visit. ^b^This diagnosis was made by otolaryngology via EGD images and physical exam. ^c^Bronchoscopy was performed by pulmonology after consultation with otolaryngology. EGD, esophagogastroduodenoscopy; ENT, ear nose throat; GI, gastroenterology

Patient	Sex	Age	EGD finding	Follow up procedure(s) by ENT	Final diagnosis
1	M	38	Right tonsillar polyp	None^a^	Left soft palate papilloma^b^
2	F	70	Enlarged tonsils and possible small ulcers	Flexible laryngoscopy	Chronic lingual tonsillitis and vascular markings on epiglottis
3	F	58	Right vocal fold lesion	Rigid laryngoscopy/stroboscopy, surgical excision	Vocal cord polyp
4	F	54	Lesion at tongue base	Flexible laryngoscopy, surgical excision	Fibroepithelial polyp
5	M	64	Interarytenoid fullness	Flexible laryngoscopy	Benign interarytenoid hypertrophy
6	F	64	Vocal cord nodule	Rigid laryngoscopy/stroboscopy	Normal anatomy
7	M	65	Possible airway foreign body	Pulmonary bronchoscopy^c^	Normal anatomy
8	F	47	Left vocal cord nodule	Flexible laryngoscopy	Normal anatomy

**Figure 1:**
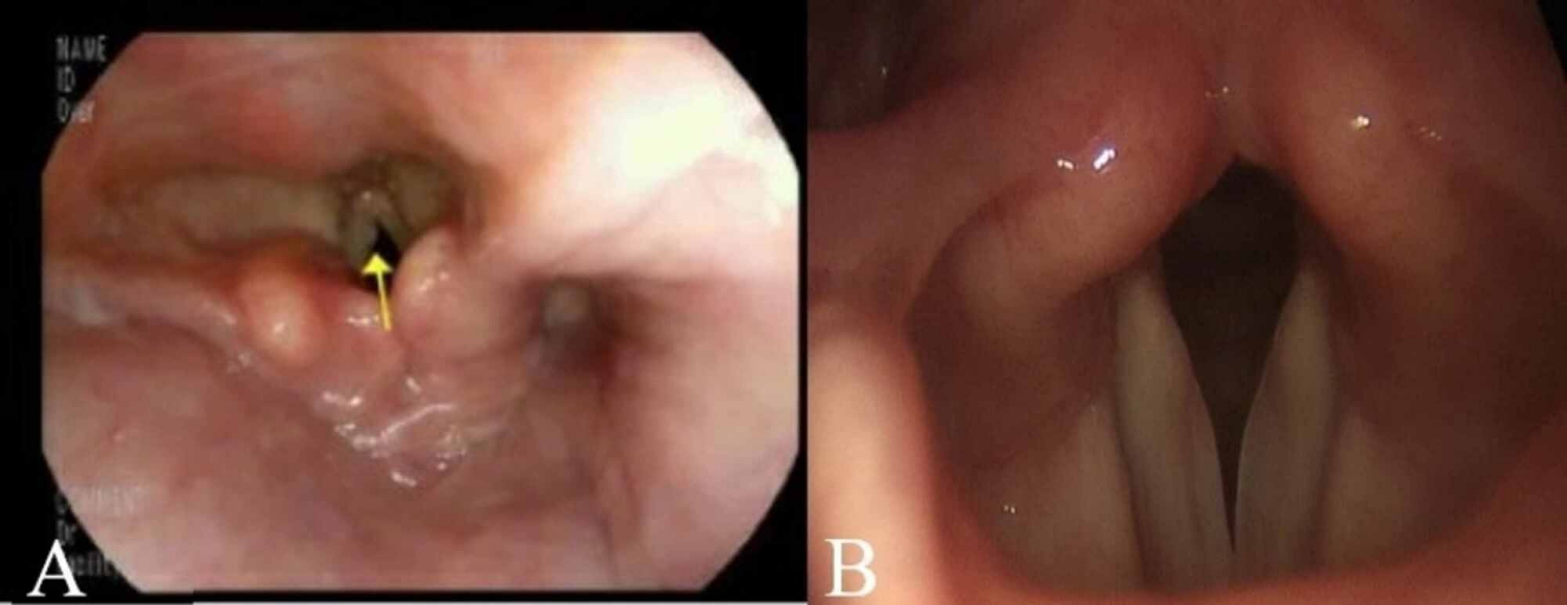
A. EGD image showing possible vocal cord nodule. B. Flexible laryngoscopy image showing normal anatomy. Images are from patient six. EGD, esophagogastroduodenoscopy

Gastroenterologists noted vocal cord nodules during EGD for 3/8 (38%) of our cases (patients three, six, and eight). Patient three was diagnosed with a vocal cord lesion via rigid laryngoscopy by otolaryngology which was subsequently surgically excised. Pathology showed a vocal cord polyp with focal mild atypia. Patients six (Figure 1A,B) and eight were determined to represent normal laryngeal anatomy after consultation and laryngoscopy by otolaryngology. 

**Figure 2:**
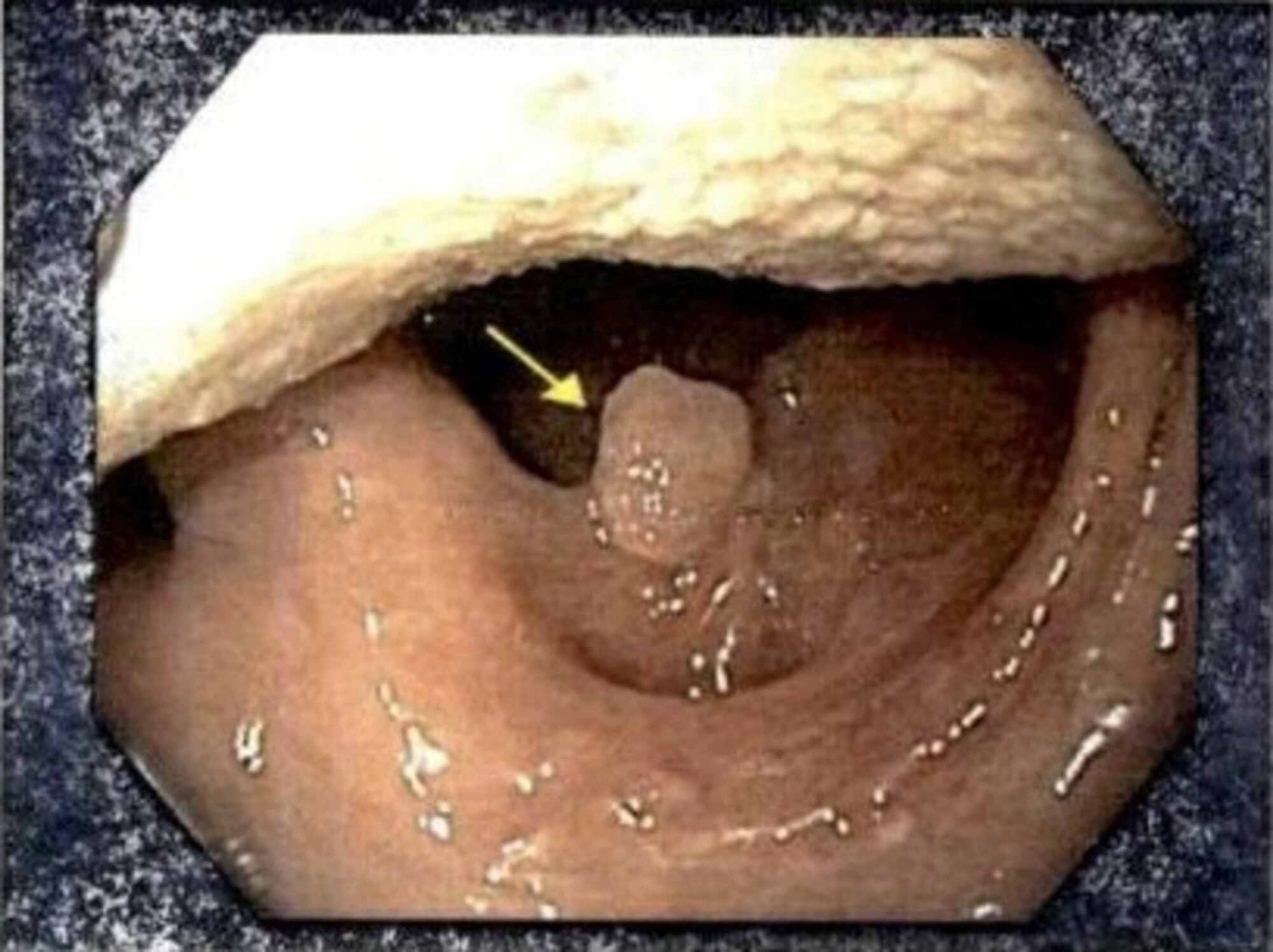
EGD image of left soft palate papilloma. Image from patient one. EGD, esophagogastroduodenoscopy

Patient one was referred for a right tonsillar polyp observed during EGD. Upon evaluation in otolaryngology clinic, the lesion was clinically diagnosed as a likely left-sided soft palate papilloma based on physical examination together with images provided by GI (Figure 2). Patient two was referred for enlarged lingual tonsils with possible small ulcers noted during EGD. Otolaryngology performed flexible laryngoscopy and found prominent vascular markings on the epiglottis. This patient was ultimately diagnosed with chronic lingual tonsillitis. The patient continues to see otolaryngology to monitor progression of these findings. Patient four was referred for a tongue base lesion found during EGD. Otolaryngology examined the lesion via flexible laryngoscopy and surgical excision was performed. Histopathologic analysis of the specimen confirmed the diagnosis of fibroepithelial polyp. 

**Figure 3:**
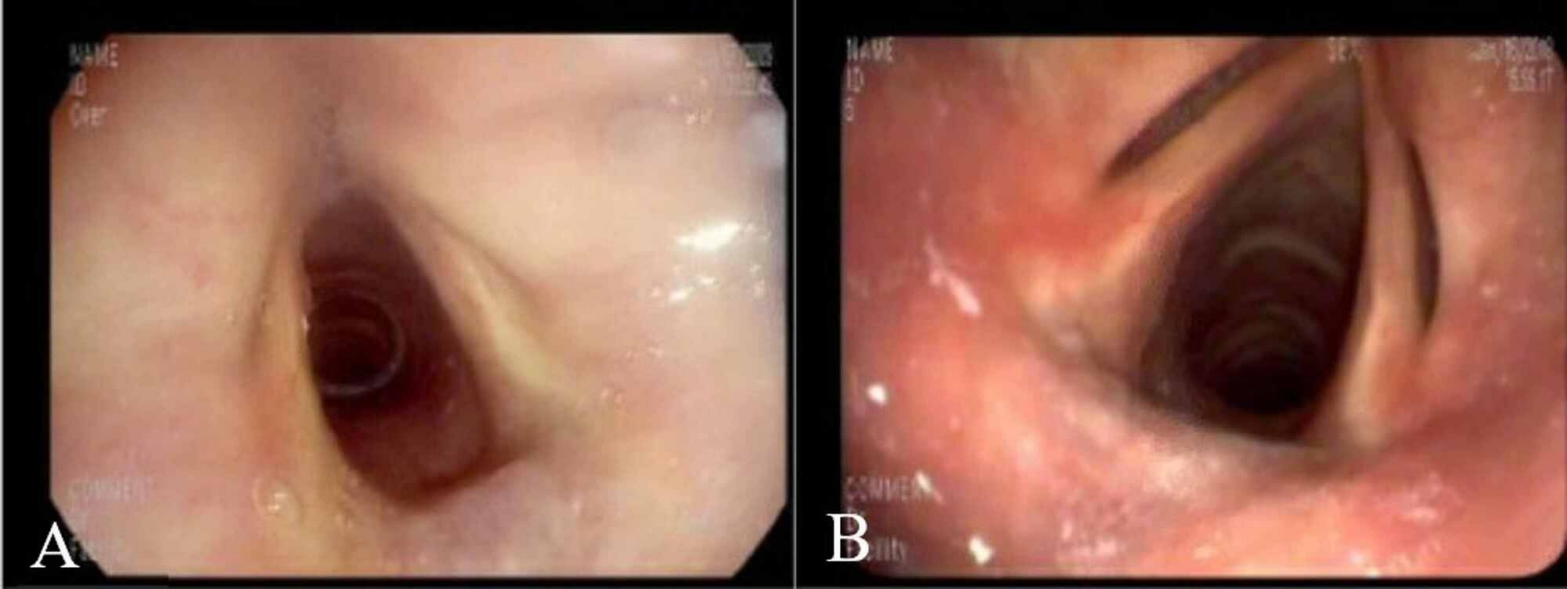
A. EGD image with possible foreign body in trachea or subglottis. B. Bronchoscopy image showing a prominent cricoid ring. Images are from patient seven. EGD, esophagogastroduodenoscopy

Patient five was referred from GI for interarytenoid fullness observed during EGD. Otolaryngology performed flexible laryngoscopy and diagnosed this patient with benign interarytenoid hypertrophy. Patient seven underwent inpatient EGD due to dysphagia during which a possible foreign body was observed in the trachea. Bronchoscopy was performed by pulmonology after consultation with otolaryngology and they determined that the possible foreign body was normal cricoid cartilage (Figure 3A,B).

## Discussion

There has been a substantial increase in the number of outpatient referrals in recent years [7], making physician referrals a fundamental component of ambulatory care in the United States [8]. However, researchers have only recently begun to assess the efficacy of inter-specialty referral and communication. This case series was designed to further investigate the nature of referrals from GI to otolaryngology following EGD by analyzing common reasons for referral and final diagnosis following consultation. Most endoscopists generally assume that laryngopharyngeal cancers should be diagnosed by otolaryngologists because they occur outside of the generally accepted anatomical domain of GI [9]. In practice, there is no standardized endoscopic cancer screening performed by otolaryngologists for patients without symptoms [9] -- by extension, there is no current screening for these cancers even though these are deadly and costly malignancies [4, 10]. In the American Society for Gastrointestinal Endoscopy’s article on quality indicators for EGD, the authors ask, among several other questions for future research, whether visualization of the oropharynx should be a standard part of a routine EGD. We agree that this question deserves systematic study and hope that our case series provides further impetus [11].

In the United States, in 2020, it is estimated that there will be 12,370 new cases of laryngeal cancer with 3,750 resultant deaths and 17,950 new pharyngeal cancer cases with 3,640 deaths [12] -- the curability of these cancers depend heavily on their stage at diagnosis [1]. Laryngopharyngeal cancers are often diagnosed at late stages, where data show that 46% of oral and pharyngeal cancers present with regional metastases upon diagnosis and 18% present with distant metastases [10]. Each of these cancers are associated with high mortality rates, showing five-year survival rates for hypopharyngeal, laryngeal, and tracheal cancers at 34.6%, 60.3%, and 53.8%, respectively [10]. It is reasonable to expect that the high mortality rates relate to their late stage of disease at time of diagnosis. Patient outcomes would be expected to improve if these cancers could be diagnosed at earlier stages -- making the case for the incorporation of basic screening for these cancers during routine EGD.

The current case series provides specific examples of head and neck pathology encountered by gastroenterologists during upper endoscopy. Although limited by a small sample size of eight cases, and the fact that most of these subjects (7/8) were the patients of a single otolaryngologist working at a tertiary medical center, we show that EGD findings from multiple cases were determined to represent abnormalities upon otolaryngologic consultation. It is important to note that the gastroenterologists had not been advised to observe the pharynx and larynx during EGD, and any observation of this area was either by chance or due to personalized methods by which these gastroenterologists perform EGD. This series indicates that without training or instruction, the potential for detecting malignant, premalignant, and benign lesions upon EGD examination within the general population is apparent and should not be ignored. The most common anatomical subsites leading to otolaryngology referral in this case series were the glottis (3/8) and oropharynx (2/8) -- both sites observed endoscopically en route to the esophageal inlet. Abnormalities at more lateral subsites, such as the pyriform recesses, were not encountered in this series. This is relatively consistent with our prior study examining referral patterns in which hypopharyngeal pathology accounted for only 7% of referrals, whereas approximately one-third of referrals from GI to otolaryngology were for oropharyngeal pathology [13]. 

Due to the relatively low incidence of laryngopharyngeal cancers compared to other malignancies, a standardized screening protocol involving laryngoscopy is likely not feasible. Instead, basic screening could be performed during the millions of EGDs performed annually [5]. Importantly, a study by Stevens et al. (2015) has shown that evaluation of the larynx and hypopharynx during EGD can be accomplished both safely and quickly [1]. After a brief training session, the participating gastroenterologists were asked to evaluate the laryngopharyngeal area prior to performing EGD and their results showed that this initial evaluation had no negative effect on the EGD exam and only added 35.8 s to the overall procedure time. Various abnormalities were noticed during EGD and subsequently referred to otolaryngology [1]. In contrast to that study, which was performed on a predominantly male VA population and involved gastroenterologists who were trained to examine the upper aerodigestive tract, our case series included slightly more women than men, and cases came from gastroenterologists who did not receive external prompting. We therefore feel that our work is complementary.

## Conclusions

Otolaryngologists and gastroenterologists are endoscopic specialists who share an interest in the upper digestive tract. Improved inter-specialty communication and combined educational initiatives may be warranted to address potential knowledge gaps between these two specialties. Our case series demonstrates that attentive gastroenterologists can detect head and neck pathology and that routine examination of the larynx and hypopharynx during EGD could increase early detection of head and neck cancers and premalignant lesions. 
